# Quantitative RT-PCR profiling of the Rabbit Immune Response: Assessment of Acute *Shigella flexneri* Infection

**DOI:** 10.1371/journal.pone.0036446

**Published:** 2012-06-04

**Authors:** Pamela Schnupf, Philippe J. Sansonetti

**Affiliations:** 1 Unité de Pathogénie Microbienne Moléculaire, Institut Pasteur, Paris, France; 2 INSERM U786, Institut Pasteur, Paris, France; University of Osnabrueck, Germany

## Abstract

Quantitative reverse transcription PCR analysis is an important tool to monitor changes in gene expression in animal models. The rabbit is a widely accepted and commonly used animal model in the study of human diseases and infections by viral, fungal, bacterial and protozoan pathogens. Only a limited number of rabbit genes have, however, been analyzed by this method as the rabbit genome sequence remains unfinished. Recently, increasing coverage of the genome has permitted the prediction of a growing number of genes that are relevant in the context of the immune response. We hereby report the design of twenty-four quantitative PCR primer pairs covering common cytokines, chemoattractants, antimicrobials and enzymes for a rapid, sensitive and quantitative analysis of the rabbit immune response. Importantly, all primer pairs were designed to be used under identical experimental conditions, thereby enabling the simultaneous analysis of all genes in a high-throughput format. This tool was used to analyze the rabbit innate immune response to infection with the human gastrointestinal pathogen *Shigella flexneri*. Beyond the known inflammatory mediators, we identified IL-22, IL-17A and IL-17F as highly upregulated cytokines and as first responders to infection during the innate phase of the host immune response. This set of qPCR primers also provides a convenient tool for monitoring the rabbit immune response during infection with other pathogens and other inflammatory conditions.

## Introduction

The New Zealand white rabbit (*Oryctolagus cuniculus*) is a widely used animal model for basic research of human diseases and pathogens, translational biomedical research and the development of vaccines or therapeutics. Rabbits are phylogenetically more closely related to primates than rodents are and have an anatomy and physiology that more closely resembles that of primates. In many instances this leads to a more acurate modeling of human conditions. In addition, the rabbit shares many advantages with rodents such as their small size, short gestation time, large litter sizes, ease of breeding and maintaining colonies, as well as the relatively low cost to purchase and house them.

The rabbit has been used as a model organism to study a number of human diseases such as cancer [1], [2] atherosclerosis [3], Alzheimer’s [4] as well as serving as an important model for eye research [5]. Within the infectious disease field, rabbit models have been established for a large number of pathogens including protozoa, such as *Schistosoma mansoni* [6] and *Entamoeba histolytica* [7]; viruses, such as the papilloma virus [8], Herpes simplex virus [9] and rotavirus [10]; fungi, such as *Candida albicans* [11] and *Aspergillus flavus* [12]; and a large number of bacteria, including enterotoxic *Escherichia coli*, *Vibrio cholerae*, *Mycobacterium tuberculosis*, *Borrelia burgdorferi*, *Chlamydia pneumoniae*, *Salmonella enterica*, *Campylobacter jejuni, Bacillus anthracis* and *Shigella flexneri* [13], [14], [15], [16], [17], [18]. These pathogen-rabbit models include ocular, dermal, visceral, pulmonary, systemic and intestinal diseases.

A hallmark of infection is the activation of the host immune response to combat the intruder. The use of the rabbit model is, however, hampered by the limited availability of species-specific products, such as antibodies. For now, the host response may be analyzed by measuring production of the limited number of cytokines for which an antibody or a functional assay is available, assaying for enzyme activity, such as MPO from neutrophils, monitoring transcriptional changes using quantitative PCR (qPCR), and histological scoring of morphological changes of the tissue and infiltration of leukocytes and lymphocytes. Indeed, due to the sparse availability of reagents, histological examination remains a gold standard for the analysis of the host immune response. However, quantitation of this response is highly laborious as it involves embedding samples, sectioning, staining and manually measuring morphological changes or cell infiltrates. In addition, early events of immune activation that precede gross morphological changes are difficult if not impossible to identify [19]. There therefore is a need to develop tools to more easily assess the immune response of the host in a quantitative manner.

Quantitative RT-PCR is a sensitive and rapid means to assay the host response to infection and, as microarrays, macroarrays or other arrays are not available for the rabbit, remains the method of choice for transcriptional analysis for the rabbit. In addition, qPCR offers a cost-effective and simple way to analyze specific target genes of interest using widely available qPCR machines [20]. While qPCR lacks the large-scale throughput of microarrays, it is considered the gold standard for gene expression analysis due to its ease of use, high detection sensitivity and wide linear dynamic range. Currently, the two most popular qPCR techniques are the TaqMan and SYBR^®^ Green technologies. While TaqMan^®^ uses gene-specific fluorescent probes, the SYBR technology utilizes a dye that intercalates into double-stranded DNA and allows the use of a single reagent with many different primer pairs, thereby also reducing running costs. The number of genes available for analysis in the rabbit has, thus far, been limited to cloned transcripts as the rabbit genome remains unfinished. In addition, the optimal amplification conditions for primers designed to popular targets are not uniform, generally cannot be run together, and are not optimized for newer qPCR machines [21]. Now, increasing coverage of the rabbit genome has permitted the prediction of more rabbit genes including those that code for cytokines (such as IL-17A, IL-17F, IL-21, and IL-22) that recently were discovered to be highly relevant in the innate immune response to pathogen challenge and in a number of inflammatory diseases. We therefore sought to increase the number of available rabbit target genes for qPCR analysis and to standarize the amplification conditions to allow simultaneous analysis of all targets. As a test system, we assessed the inflammatory response elicited by infection of rabbits with *Shigella flexneri*.

*Shigella* is a gram-negative facultative anaerobic and intracellular human pathogen and the causative agent of bacillary dysentery or shigellosis [22]. *Shigella* infects via the fecal-oral route, crosses the colonic mucosa via M-cells and is taken up by macrophages and dendritic cells. *Shigella* escapes these immune sentinels by inducing a pro-inflammatory cell death called pyroptosis and invades the epithelial layer basolaterally to replicate and spread cell-to-cell to neighboring cells via actin-based motility [22]. The *Shigella flexneri*-rabbit ileal loop model is commonly used to perform *Shigella* vaccine research, study the interaction of *Shigella* with the intestinal barrier, investigate the induction of the innate immune response and evaluate the virulence of *S. flexneri* mutants. Infection of rabbit ligated ileal loops with *S. flexneri* leads to a strong inflammatory response that is characterized by bacterial invasion of the epithelium, massive recruitment of polymorphonuclear cells and extensive destruction of the epithelial lining at its acute phase [19]. This animal model thereby accurately recapitulates the symptoms seen in rectal biopsies of humans suffering from shigellosis [19], [23]. However, during shigellosis, *Shigella* infection foci are focally distributed [24]. This renders detection of affected areas by histology difficult even in severely sick patients and requires several biopsies to obtain a representative analysis [23]. Although shigellosis in the rabbit is robust, this issue also arises in this model, making a quantitative analysis using relatively large pieces of intestine an important complementary method to histological analysis of thin sections.

We hereby report the design of a set of twenty-four primer pairs for simultaneous qPCR analysis to highly relevant rabbit immune genes for a rapid, sensitive and quantitative analysis of the rabbit immune response. This tool was able to clearly detect the activation of the innate immune system at early stages of *Shigella* infection before easily visible morphological alterations had taken place within the intestinal mucosa.

## Materials and Methods

### Bacterial Propagation and Infection

The streptomycin-resistant invasive wild-type *Shigella flexneri* strain M90T-Sm of serotype 5a and its virulence plasmid-cured non-invasive derivative BS176 were thawed from –80°C stocks, grown on Tryptic Soy (TCS) Agar-Congo red (CR) plates (3% BBL-trypticase soy agar (BD Biosciences), 0.1% CR) for 16 hrs at 37°C and kept at 4°C. For infection, one colony of each strain from freshly streaked plates was grown in TCS medium at 37°C for 16 hours with agitation two days before the experiment and kept at room temperature for 8 hrs the next day. In the evening before the experiment, 50 µl of culture of each strain was plated onto TCS plates (without CR) and incubated at 37°C for 16 hrs to form a bacterial lawn. The morning of the experiment, physiological saline (0.9% NaCl) was added to each plate and plates were scraped to resuspend the bacteria. Bacterial suspensions were diluted to OD_600_ = 4 (equivalent to 3×10^9^ bacteria ml^−1^). As a control, the bacterial suspension was diluted by 10^6^ fold and 25 µl were plated in triplicate on TCS-CR plates. After incubation at 37°C for 16 hrs, bacterial colonies were enumerated (approximately 75 colonies are expected) and checked for proper colony morphology (M90T colonies appear red and are smaller than those of BS176).

### Rabbit Illeal Loop Infections

The rabbit illeal loop model protocol was approved by the Comite Regional d’Ethique pour l’Experimentation Animale in Paris 1 (protocol #20070004). Surgery was performed essentially as described previously with minor modifications [19]. Twelve male New Zealand White rabbits weighing 2.4–2.6 kg (Charles River Laboratories, St. Aubin, France) were split into 2 groups for short (4 to 5.5 hrs) and long (8 hrs) time points of infection. Animals were received 10 days prior to surgery and treated with Mucoxid (2.8 g L^−1^ sodium sulfadimethoxine salt in the drinking water) (CEVA Sante Animale, France) until the day prior to surgery to minimize infection with *Coccidia* parasites. Three independent experiments using two rabbits each for early and late time points were performed. Rabbits were fasted 24 hrs before infection, sedated intravenously by ear vein injection with 0.05 ml kg^−1^ Calmivet (Vétoquinol) containing 0.5% acepromazine and anesthesized by the same route with 0.2 ml kg^−1^ of Imalgene® 1000 (containing 10% ketamine HCl) (Merial, France). Prior to laparotomy, 2 ml Xylovet (CEVA Sante Animale, France) containing 2.1% lidocaine, was injected intradermally in the abdomen along the site of incision. The small intestine was exteriorized and the cecum was localized. Twelve loops of five cm segments of ileum starting at the ileum-cecum transition were ligated, avoiding all Peyer’s patches, while maintaining the existing vasculature. Every other loop was injected with 0.5 ml of bacterial suspension or the saline control using a 26-gauge needle. The injection order of control, wild-type and avirulent *Shigella*-injected loops were randomized for each rabbit and performed in duplicate within an animal. Loops were returned into the abdominal cavity, the abdomen was closed and the animals were returned to their cage for 4–8 hrs. Animals were sacrificed by intravenous injection of 120 mg kg^−1^ sodium pentobarbital (Doléthal, Vétoquinol, France). The exudate of each loop was suctioned using an 18-gauge needle and measured before loops were dissected and processed for RNA extraction and histology.

### Tissue Preparation, RNA Extraction and cDNA Synthesis

For immunohistochemical staining of *Shigella*, 1–2 cm-long pieces of rabbit ileal loop were fixed for 2 days at 4°C in 10 ml of 4% paraformaldehyde in phosphate-buffered saline, embedded in paraffin and sectioned into 5 µm sections (3–4 sections/slide) using a microtome. Sections were deparaffinated, rehydrated and split into antibody control and test groups. Sections were permeabilized for 15 min with 0.1% Triton-X100, treated with 3.3% H_2_O_2_ for 15 min and washed. Samples were blocked for 30 min with Ultra V block (Lab Vision Corp; Thermo Fisher) and incubated overnight with an in-house mouse polyclonal anti-*S. flexneri* serotype 5 serum or without serum as a control. Samples were then incubated with horseradish peroxidase conjugated anti-rabbit antibody (K4002, DAKO) for 1 hr, revealed with 3-amino-9-ethylcarbzole (AEC+, K3461, DAKO), counterstained with hematoxylin (Thermo Shandon) and mounted with aqueous mounting medium (Merck).

For RNA extraction, 1 cm long tissue samples were immediately submerged in 4 ml Trizol (Invitrogen), homogenized for 1 min with a tissue homogenizer and stored at –80°C until further processed. A volumen of 1.2 ml of each sample was spun at 4°C for 15 min at 12,000×g to remove debris and DNA, 1 ml of supernatant was mixed with 200 µl chloroform, shaken for 154 seconds, incubated at RT for 2–3 minutes and spun for 10 min at 12,000×g at 4°C. RNA was precipitated by adding 500 µl of the aqueous phase to an equal volume of isopropanol and spun at 14,000×g at RT for 10 min. RNA was washed with 75% ethanol, spun at 14,000×g at 4°C for 10 min, dried and resuspended in 60 µl DEPC-treated H_2_O (Ambion). RNA was quantitated on a spectrophotometer (Nanodrop 2000) and 40 µg of RNA was used for a second RNA purification using the Nucleospin RNA II kit (Macherey-Nagel GmbH) including the on-column digestion of DNA. The final RNA concentration was determined using a spectrophotometer (Nanodrop 2000) and the purity was assessed by agarose gel electrophoresis or the Agilent’s 2100 Bioanalyzer.

CDNA synthesis was performed on 3 µg of RNA in a 10 µl sample volume using SuperScript II reverse transcriptase (Invitrogen) as recommended by the manufacturer. The RNA was incubated with 0.5 µg of oligo(dT)12–18mers primers (Invitrogen) for 7 min at 70°C and then transferred onto ice. Then, 9 µl of a master mix containing 4 µl of SuperScript II buffer, 2 µl of 0.1 M DTT (Invitrogen), and 1 µl each of dNTPs stock (10 mM) (Invitrogen), Rnasin (40 UI) (Promega) and SuperScript II (Invitrogen) were added to the RNA sample, spun and incubated at 42°C for 60 min followed by 5 min at 70°C to inactivate the enzyme. CDNA was stored at −20°C.

### Primer Design and Quality Control

Primers were designed using the online program Primer3Input (www.fokker.wi.mit.edu/primer3/input.htm). Primer selection parameters were set to primer size: 20–26 nts; primer melting temperature: 62 to 64°C; GC clamp: 1; and product size range: generally 120–140 bp but down to 80 bp if no appropriate primers could be identified. Primers were ordered from Invitrogen and tested using serial 2-fold dilutions of cDNA, from 1 in 40 to 1 in 2560, from 8 hour *S. flexneri*-infected rabbit ileal tissue to ensure efficient and comparable linear amplification of the amplicons across a wide range of target abundance.

### Cytokine Expression Analysis by Quantitative Real-time PCR

QRT-PCR was performed in a total volume of 15 µl including 300 ng of cDNA, primers (0.2 µM each) and 7.5 µl of Power SYBR Green mix (Applied Biosystems). Reactions were run in duplicate on an ABI 7900HT (Applied Biosystems) using the universal thermal cycling parameters (2 min 60°C, 95°C 10 min, 40 cycles of 15 sec at 95°C and 60 sec at 60°C; dissociation curve: 15 sec at 95°C, 15 sec at 60°C and 15 sec at 95°C). Results were obtained using the sequence detection software ABI 7900HT SDS2.2 and analyzed using Microsoft Excel. For all samples, dissociation curves were acquired for quality control purposes. In addition, amplification products were visualized by agarose gel electrophoresis. For gene expression quantification, we used the comparative Ct method. First, gene expression levels for each sample were normalized to the expression level of the housekeeping gene encoding Glyceraldehyde 3-phosphate dehydrogenase (GAPDH) within a given sample (Δ -Ct); the difference between the infected loops compared to the control uninfected (saline only) loop was used to determine the Δ -Δ-C_t_. The log_2_ Δ -Δ-C_t_) gave the relative fold increase in gene expression of the test versus the control condition. Statistical significance of the fold difference between the infected and the uninfected samples was calculated with the Wilcoxon Signed Rank test using PRISM software.

## Results

**Figure 1:**
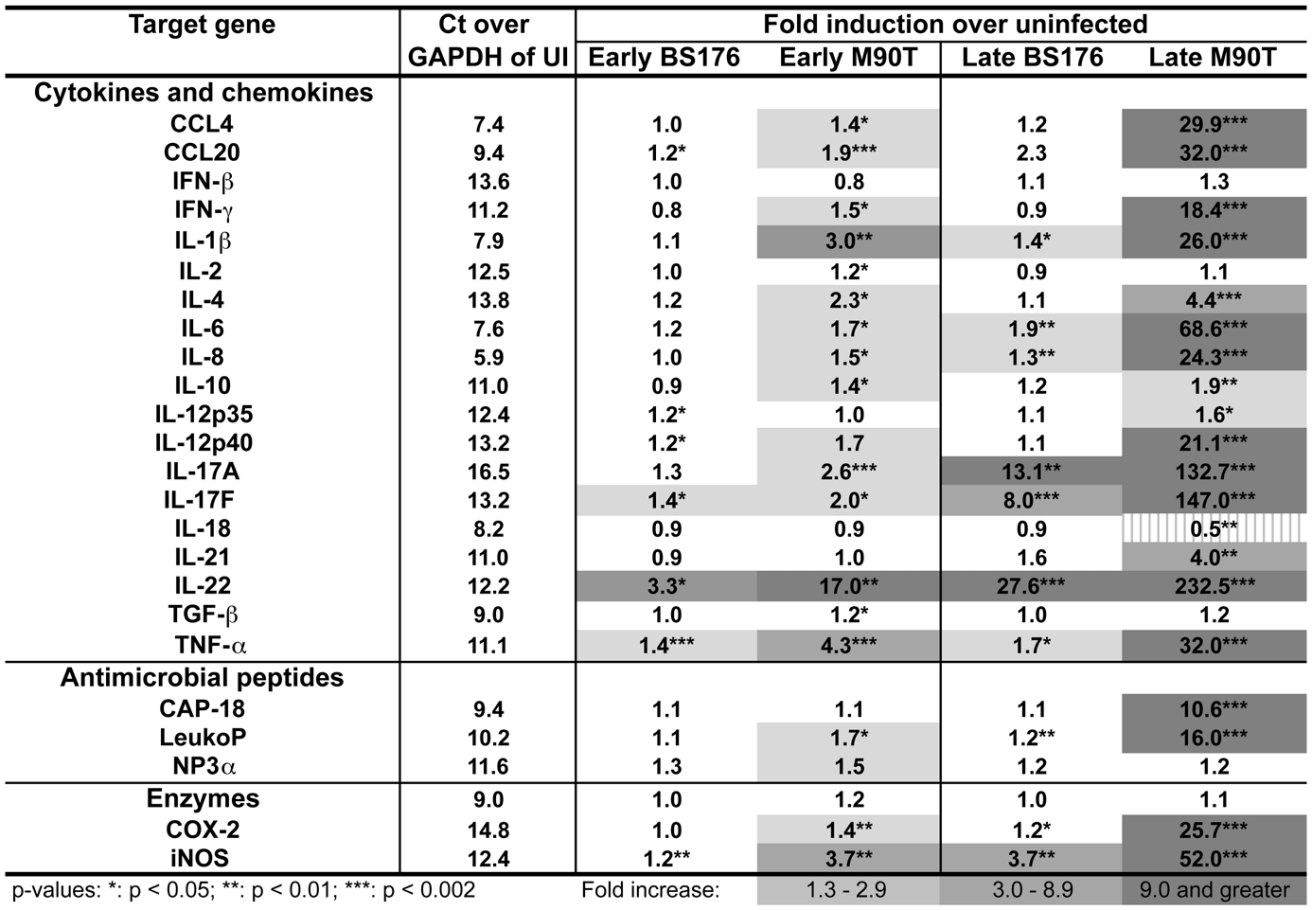
Gene expression changes in rabbit illeal loops infected with avirulent or virulent *Shigella*. Transcriptional response at A) early (4 to 5.5 hrs) and B) late (8 hrs) time points post infection with the avirulent BS176 strain or the virulent M90T strain. The fold induction in gene expression over uninfected (UI) control loops (Δ-Δ-Ct) are given as the median of twelve samples obtained from six rabbits in three independent experiments. Basal level expressions are listed as the median Ct value above the housekeeping gene GAPDH (Δ-Ct) from twelve uninfected loops at the early time point from six rabbits. As GAPDH is an abundant transcript, smaller changes in fold Ct compared to GAPDH signify more abundant gene transcripts. Increased shading highlights increasing upregulation of statistically significan values while stripes signify downregulation. The statistical significance (p-value) was calculated using the Wilcoxon Signed Rank Test.

We designed and tested twenty-four real-time PCR primer pairs for a quantitative gene expression analysis of key rabbit genes involved in the innate and adaptive immune response to infection (Table 1). We assayed the expression of three chemokines [IL-8, chemokine (C-C motif) ligand (CCL)-4, and CCL20], sixteen cytokines [Interleukin (IL)-1β IL-2, IL-4, IL-6, Il-10, IL-12p35, IL12/23p40, IL-17A, IL-17F, IL-18, IL-21, IL-22, Interferon (IFN)- β, IFN-γ, Transforming growth factor (TGF)-β and Tumor necrosis factor (TNF)-α], three antimicrobials [Leukocyte Protein (LeukoP) p15, neutrophil defensin NP-3α, and the cathelicidin CAP-18] and two enzymes [inducible Nitric oxide synthase (iNOS) and cyclo-oxygenase (COX)-2]. This set of twenty-four genes exhibited a wide range of basal expression levels in uninfected loops (5.9 to 14.8 fold Ct over the expression level of the housekeeping gene GAPDH) (Figure 1). The most abundant transcript at the steady state level was IL-8, followed by CCL4, IL-6, IL-1β and IL-18, while rather low levels were detected for IL-17A, COX-2, IL-4, IFN-β, IL-12/23p40, IL-12p35, IL-2, iNOS and IL-22.

**Table 1:** Rabbit primer pairs for quantitative RT-PCR analysis.

Rabbit target gene	Forward and reverse primer sequences	Primer location within CDS	Target size	NCBI Accession #
**Control gene**				
GAPDH	PS182:TGACGACATCAAGAAGGTGGTG; PS183:GAAGGTGGAGGAGTGGGTGTC	Exon 1 of 1	120 nts	NM_001082253
**Cytokines & Chemokines**				
CCL4	PS343:GAGACCACCAGCCTCTGCTC; PS344:TCAGTTCAGTTCCAAGTCATCCAC	Exon 2 and 3 from 3	123 nts	NM_001082196
CCL20	PS565:TATCGTGGGCTTCACACAGC; PS566:CCATTCCTTCTTCGGATCTGC	Exon 2 and 3 from UD	115 nts	Trace Archive
IFN-β	PS291:TCCAACTATGGCACGGAAGTCT; PS292:TTCTGGAGCTGTTGTGGTTCCT	Exon 1 from 1	133 nts	XM_002707968
IFN-γ	PS186:TGCCAGGACACACTAACCAGAG; PS187:TGTCACTCTCCTCTTTCCAATTCC	Exon 1 and 2/3 from 4	127 nts	NM_001081991
IL-1β	PS168:TTGAAGAAGAACCCGTCCTCTG; PS169:CTCATACGTGCCAGACAACACC	Exon 3/4 and 4 from∼6	128 nts	NM_001082201
IL-2	PS275:GCCCAAGAAGGTCACAGAATTG; PS276:TGCTGATTGATTCTCTGGTATTTCC	Exon 2/3 and 3 from 4	128 nts	NM_001163180
IL-4	PS267:CGACATCATCCTACCCGAAGTC; PS268:CCTCTCTCTCGGTTGTGTTCTTG	Exon 1 and 2/3 from 4	122 nts	NM_001163177
IL-6	PS170:CTACCGCTTTCCCCACTTCAG; PS171:TCCTCAGCTCCTTGATGGTCTC	Exon 2 from ∼5	135 nts	NM_001082064
IL-8	PS287:CCACACCTTTCCATCCCAAAT; PS288:CTTCTGCACCCACTTTTCCTTG	Exon 2 and 3 from 4	122 nts	NM_001082293
IL-10	PS281:CTTTGGCAGGGTGAAGACTTTC; PS282:AACTGGATCATCTCCGACAAGG	Exon 1 and 3 from 5	126 nts	NM_001082045
IL-12p35	PS214:AAGGCCAGACAAACTCTAGAATTC; PS215:TTGGTTAACTCCAGTGGTAAACAGG	Exon 3/4 and 4/5 from ∼8	116 nts	XM_002716291
IL-12/IL-23p40	PS211:CTCCGAAGAAGATGGCATTACC; PS212:TCTCCTTTGTGGCAGGTGTATTG	Exon 2 from 6	126 nts	XM_002710347
IL-17A	PS591:CCAGCAAGAGATCCTGGTCCTA; PS592:ATGGATGATGGGGGTTACACAG	Exon 3 from 3	112 nts	XM_002714498
IL-17F	PS589:AAAATCCCAAAGTGGAGGATGC; PS590:AGCGGTTCTGGAAGTCATGTGT	Exon 2 from 3	138 nts	XM_002714499
IL-18	PS575:ACCAAGGACAGCAACCTGTGTT; PS576:ACAGAGAGGCTTACAGCCATGC	Exon 3 and 4 from 5	120 nts	NM_001122940
IL-21	PS579:GCTGGCAACATGGAAAGGATAG; PS580:TTGCCCTTTGGAGCTTGATTTA	Exon 4 from 8	84 nts	XM_002717257
IL-22	PS567:ACCTCACCTTCATGCTGGCTAA; PS568:CATGGAACAGCTCATTCCCAAT	Exon 1 and 2 from 5	84 nts	XM_002711248
TGF-β	PS199:CAGTGGAAAGACCCCACATCTC; PS200GACGCAGGCAGCAATTATCC	Exon 6 and 7 from ∼8	140 nts	NM_001082660
TNF-α	PS174:CTGCACTTCAGGGTGATCG;PS175:CTACGTGGGCTAGAGGCTTG	Exon 1 and 3 from ∼4	133 nts	NM_001082263
**Antimicrobials**				
CAP-18	PS176:CCCAAGAGTCCCCAGAACCTAC; PS177:TCTGTCCTGGGTGCAAGTTTC	Exon 3/4 and 4 from ∼4	130 nts	NM_001082305
LeukoP	PS225:GTCGCCGTCTGAGATATGAGGA; PS226:GTTGAGTGGGATCCTGGATTTG	Exon 1 and 2 from 2	140 nts	NM_001082325
NP3α	PS205:ACCTTACAGGGGAGGAAAGCTC; PS206:GTACATAGCGGGCTCCATTGAC	Exon 1 and 2 from 2	132 nts	NM_001082298
**Enzymes**				
COX-2	PS329:CGGATTCTACGGTGAAAACTGC; PS330:GACGATGTTCCAGACTCCCTTG	Exon 1 and 2 from 10	124 nts	NM_001082388
iNOS	PS573:GACGTCCAGCGCTACAATATCC; PS374:GATCTCTGTGACGGCCTGATCT	Undetermined	102 nts	XM_002718780

Rabbit primer pairs were designed within the coding sequence (CDS) of rabbit genes identified either by homology to the mouse or human gene using the NCBI rabbit.

Trace Archive database, or predicted or experimentally-determined CDSs available at NCBI. All primers were designed using identical design parameters (see Materials and Methods) and made to span exon junctions when possible.

CAP: cationic animicrobial protien (a cathelicidin (LL-37 in humans); CCL: cheomokine (C-C motif) ligand; COX: cyclo-oxygenase; iNOS: inducible Nitric oxide synthase; IFN: Interferon; IL: Interleukin; LeukoP: Leukocyte protein (cationic antimicrobial peptide); NP: Neutrophil protein (a defensin); TGF: Transforming growth factor.

We then used the established rabbit ileal loop model of shigellosis [19] to characterize the host response to acute *S. flexneri* infection at both early (4 to 5.5 hrs) and late (8 hrs) time points after infection. For each time point, six rabbits from three independent experiments were utilized. Duplicate ileal loops for each rabbit were injected with the virulent, invasive *S. flexneri* strain M90T, the avirulent and noninvasive *S. flexneri* strain BS176, and physiological saline alone for a total of twelve samples per time point for each condition. At indicated times after infection, animals were sacrificed and portions of the ligated loops were processed for RNA extraction and histology.

**Figure 2:**
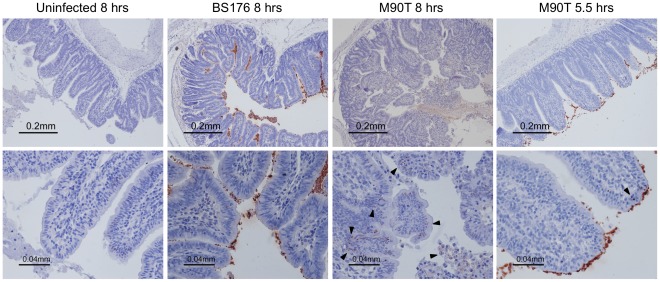
Immunohistochemistry of rabbit ileal loops left uninfected or infected with *Shigella* strains. Tissue sections of rabbit illeal loops taken at either 4–5.5 or 8 hours post surgery were stained for *Shigella* (brown) with a murine polyclonal anti-*S. flexneri* 5a LPS serum and counterstained with hematoxylin. Representative images are shown. Images are taken at 100x (top) and 400x (bottom) magnification. Destruction of villi and intracellular localization of the invasive strain M90T can clearly be seen only by 8 hrs and not by 5.5 hrs, whereas clear morphological changes were not observed even after 8 hrs of infection with the avirulent BS176 strain. Arrows point to epithelial cells that also stain for virulent *S.* *flexneri*.

At the early time point of infection (4 to 5.5 hrs), the avirulent strain did not induce any structural changes in the epithelium (data not shown), while only minor morphological changes in the intestinal structure and invasion of the villi could be detected by histology for the virulent strain by 5.5 hours (Figure 2). Villi were slightly swollen and showed early signs of leukocyte infiltration. In contrast, villi destruction, leukocyte infiltration and intracellular bacterial localization were clearly visible for the virulent strain by 8 hours of infection. Loops infected with the avirulent strain for 8 hours showed no apparent morphological alterations as compared to the non-infected control (Figure 2).

**Figure 3:**
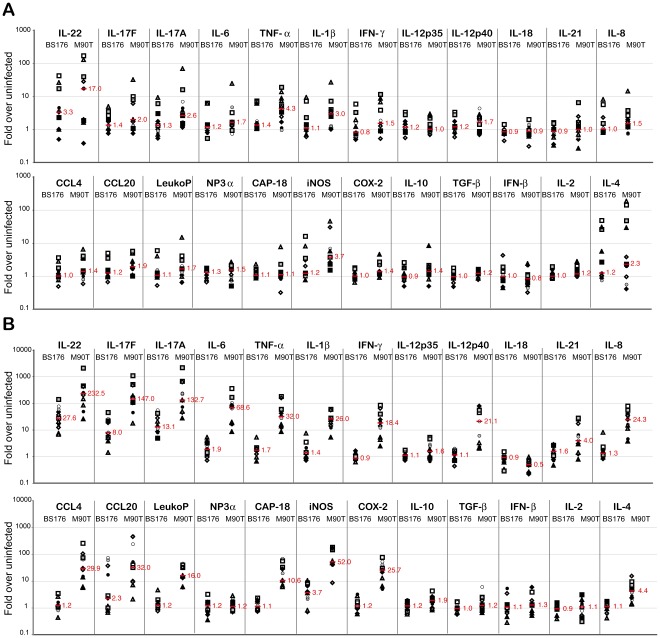
Gene expression changes in rabbit ileal loops infected with avirulent or virulent *Shigella*. Transcriptional response at A) early (4 to 5.5 hrs) and B) late (8 hrs) time points post infection with the avirulent BS176 strain or the virulent M90T strain. The median fold induction over uninfected controls for each gene is indicated in red while symbols represent values from individual loops. A total of twelve loops from six animals obtained in three independent experiments are shown. Note the difference in scale for the early and late time points.

The gene expression profile at early times of infection with virulent *S. flexneri* revealed a substantial increase in pro-inflammatory cytokines and an early antimicrobial response as compared to uninfected (UI) control loops (Figure 1). This early response was dominated by IL-22, TNF-α, iNOS and IL-1β expression (3.7 to 17 fold increase), while IL-17A, IL-17F and IL-4 expression were strongly induced in some but not all samples (Figure 3A). Indeed, infection with the virulent strain also led to an increase in expression (1.5 to 2.3 fold) for most of the other pro-inflammatory mediators tested (CCL20, IL-6, IL-12p40, and IFN-γ) as well as to an upregulation (1.7 fold) of the antimicrobial LeukoP as compared to the UI control loops. Infection with avirulent *S. flexneri* led to a substantial upregulation of only IL-22 (3.3 fold), although upregulation (1.4 fold) could also be detected for IL-17F and TNF-α.

After 8 hours of infection, at the more acute phase, the virulent strain had induced an extensive increase in many cytokines, particularly IL-22, IL-17A and IL-17F (132 to 233 fold) (Figure 2). These cytokines were also highly upregulated (8 to 28 fold) by infection with the avirulent strain. While the expression of many pro-inflammatory cytokines (IL-6, TNF-α, IL-1β, IFN-γ and IL12p40) was also strongly induced (18–69 fold) by the virulent strain, these cytokines showed either no increase (IFN-γ and IL12p40) or only slightly elevated levels (1.4 to 1.9 fold) for the avirulent strain. Interestingly, infection by virulent *S. flexneri* also led to a significant decrease (2.0 fold, p<0.01) in IL-18 at the late time point (Figure 1 and Figure 3B). Furthermore, all chemoattractants (IL-8, CCL4 and CCL20) showed large increases (24 to 32 fold) in expression in loops infected with virulent *S. flexneri,* while remaining mostly unresponsive when challanged with the avirulent mutant, although some samples showed high CCL20 expression (Figure 3B). Likewise, the antimicrobials CAP-18, LeukoP and iNOS showed a strong induction (11 to 52 fold) after infection with the virulent strain at the late time point while all, except iNOS (3.7 fold increase), remained largely at the basal level during challenge with the avirulent strain. The potent pro-inflammatory response elicited was not offset by an increased abundance in anti-inflammatory cytokine transcripts as TGF-α expression remained largely unchanged under all conditions tested and IL-10 was only slightly (1.9 fold), albeit significantly (p<0.01), increased by the virulent strain after 8 hours of infection. IL-4 expression continued to increase over time (from 2.3 to 4.4 fold) for the wild-type strain but remained unchanged for the avirulent mutant while IL-21 transcript showed a significant increase (4.0 fold) only at 8 hours post infection with the virulent strain. Finally, IL-2 and IFN-β remained largely unchanged in all conditions tested.

We hereby identified four classes of gene expression profiles: i. those that are highly upregulated only by infection with virulent *S. flexneri* (CCL4, CCL20, IFN-γ, IL-1β, IL-4, IL-6, IL-8, IL-12/23p40, IL-21, TNF-α, CAP-18, LeukoP and COX-2); ii. those that are down-regulated by infection with virulent *S. flexneri* (IL-18); iii. those that are highly upregulated by infection of both the virulent and avirulent strains (IL-22, IL-17A, IL-17F and iNOS); and iv. those that remain largely unmodulated by infection with either the avirulent or the virulent strain (IL-2, Il-12p35, TGF-β and IFN-β). In addition, our results point to IL-22, IL-17A and IL-17F as key innate responders to bacterial challenge in the intestine. Indeed, infection with virulent *Shigella* induced a rapid and strong (133 to 233 fold) induction by 8 hours while the eight next most highly expressed cytokines averaged 32 fold (18 to 69 fold) induction. Interestingly, a significant induction (8 to 28 fold) of IL-22, IL-17A and IL-17F was also observed for the avirulent strain, while induction of the eight next most expressed cytokines during infection with the virulent strain were either not induced or induced weakly (<2-fold).

## Discussion

*Shigella flexneri* infection in humans leads to an acute intestinal infection with symptoms ranging from watery diarrhea to severe bloody mucoid diarrhea accompanied by fever and intestinal cramps [25]. Immunohistological analysis of rectal biopsies from *Shigella*-infected patients revealed extensive synthesis of a number of proinflammatory cytokines (IL-1α, IL-1β, IL-6, TNF-α and IFN-γ) at the local site of infection during the acute phase and increasing frequencies of cytokine-producing cells correlated with increasing severity of the disease. In addition, *Shigella* also induced the local production of a large number of other cytokines (TNF-α, IL-4, IL-10, TGF-β, IL-1ra and IL-8) [23]. Marked inflammation was accompanied by infiltration of granulocytes, T lymphocytes, macrophages and natural killer (NK) cells [26]. As seen during the natural infection, *Shigella* infection of rabbit ileal loops led to a large increase in mRNA abundance for the pro-inflammatory cytokines IL-1β, TNF- α, IL-6, IL-4, IFN-γ and IL-8, highlighting the similar cytokine response elicited in the two systems. In addition, we observed a large induction for IL-12/23p40 and a moderate induction of IL-21. IL-12 and IL-21 activate NK cells to promote a Th1 response while IL-23 is critical for IL-22 expression and sustaining Th17 responses.

*In vitro* studies characterizing the colonic epithelial cell response to invasive *S. flexneri* infection using microarrays revealed a strong upregulation of genes encoding chemokines (IL-8, CCL20, CXCL1 and 2), cytokines (GM-CSF and TNF-α) and adherence molecules (ICAM-1) [26] that together likely induce the strong recruitment of PMNs, NK cells, lymphocytes and dendritic cells that is observed during the natural infection of the human colon. Similarly, we observed a strong induction of mRNA abundance of the tested chemokines (CCL4, CCL20 and IL-8) and cytokines (TNF-α and others) in the rabbit model. The transcriptional regulation for IL-8 also showed high concordance with relative IL-8 protein levels detected in infected rabbits at various times after infection [27]. In the rabbit ileum, most IL-8 is produced by intestinal epithelial cells [27]; however, in human rectal biopsies of shigellosis patients, IL-8 is confined mainly to the crypt lumen while in healthy biopsies, IL-8 immunostaining gives strong labeling in the crypts [23], suggesting a localized storage depot at this location. Whether this kind of IL-8 storage is present in the rabbit ileum is unclear although it could explain the highly abundant IL-8 transcript level detected in our uninfected rabbit control loops.

The pro-inflammatory cytokine IL-18 was the only cytokine that decreased (2 fold) in expression levels in the rabbit model after infection with the virulent *Shigella* strain. IL-18 is produced by macrophages, which are efficently killed when infected with virulent *Shigella* [22]. IL-18 mRNA down-regulation was also previously observed in an IL-8 supplemented mouse model of shigellosis [28], although rectal tissues from patients infected with *S. dysenteriae* show an influx (3 fold) of IL-18-expressing cells at the acute stage compared to healthy controls [29]. As no transcriptome data is available for IL-18 in humans, these discrepant findings may be due to the method used to analyze IL-18 abundance or due to differences in the time points analyzed after the onset of disease.

For anti-inflammatory markers, we included IL-10 and TGF-β in our analysis. Human rectal biopsies from *Shigella*-infected patients had shown increased production of TGF-β and IL-10 cytokine expression as compared to healthy controls. However, our transcriptional analysis in the rabbit did not identify an upregulation for TGF-β and only a small increase (1.9 fold) was observed for IL-10 [23]. Notably, increased levels of TGF-β and IL-10 were detected at the convalescent stage for the humans while our analysis in the rabbit focuses on the early acute phase of the disease, possibly explaining the differences observed. Differences in the anatomical location may also play a role.

In addition to the extensive pro-inflammatory response elicited during infection with *Shigella*, *Shigella* was also found to strikingly down-regulate the production of the cathelicidin LL-37, the human homolog of CAP-18, in the epithelial lining of rectal biopsies from *Shigella*-infected patients [30] while LL-37 could be detected in infiltrating granulocytes and macrophages. *Shigella* could also down-regulate LL-37 and human beta defensin 3 in an *in vitro* infection models using human colonic epithelial cells [31]. As seen in the human biopsies, CAP-18 is exclusively localized to the surface epithelium in healthy rabbit rectal samples and *Shigella* infection also led to an almost complete loss of this pool with a concomittant infiltration of inflammatory cells (macrophages and neutrophils) expressing CAP-18 [32]. Notably, CAP-18 was not down-regulated in the proximal colon and its expression pattern in the ileum has not been explored. In our rabbit ileal loop model, CAP-18 transcript levels were upregulated only at the eight hour time point when abundant inflammatory cell infiltration into the villi was observed and levels of the neutrophil antimicrobial LeukoP transcript was also strongly enriched. These observations suggest that the observed increase in CAP-18 mRNA levels may be due to inflammatory cell influx.

iNOS expression was markedly upregulated even at the early time point, revealing a first responder type of response of this enzyme. These results also accurately recapitulate the natural infection. Strong iNOS upregulation in the surface epithelium and infiltration of iNOS positive cells, accompanied by increased iNOS mRNA levels, was observed in rectal biopsies from human patients with acute shigellosis but not in healthy controls [33], [34]. Furthermore, upregulation of COX-2 transcripts in the rabbit model echoes the increased prostraglandin levels observed in stool samples of shigellosis patients [34].

In addition to the previously established activation of the classical pro-inflammatory cytokines and chemokines, we observed that the early transcriptional response in rabbits infected with *Shigella* was dominated by the cytokines IL-22, IL-17A and IL-17F. Interestingly, unlike any of the other cytokines, these cytokines were quite strongly induced during infection with both the avirulent non-invasive strain as well as with the invasive wild-type *Shigella* strain. These results point to IL-22, IL-17A and IL-17F as first responders to not only pathogenic bacteria but also to changes in gut bacterial composition.

In the adaptive immune system, Th17 cells are a relatively newly discovered Th subset that produce IL-17A, IL-17F, IL-22 and IL-21 and are important for the host defense to bacterial pathogens at mucosal surfaces. Th17 cells are the primary Th cell type primed in a murine lung model of shigellosis and are important for mediating protective immunity [35]. However, in this mouse model, IL-17A and IL-22 could be detected above uninfected controls only by 6 days after infection. In the rabbit illeal loop model, the rapid induction (4 to 8 hrs) of IL-22, IL-17A and IL-17F is incompatible with Th17 stimulation and points to IL-17 and IL-22 production by innate immune cells. Indeed, the IL-22-IL-17 axis of innate immune cells is a very recent, yet rapidly expanding, field of research [36], [37], [38]. IL-17A and IL-17F are able to induce proinflammatory cytokines, chemokines and antimicrobial peptide expression, as well as to promote neutrophil recruitment [39], [40] and provide mucosal host defenses against fungal and bacterial infections [39], [41]. As with *Shigella*, rapid production (4 to 24 hours) of IL-17A by the host was observed after infection with a number of bacterial pathogens (*S. enterica* serovar Typhimurium in the intestine, *L. monocytogenes* in the liver, *Klebsiella pneumoniae* in the lung and with *Escherichia coli* after intraperitonneal challenge) [42], [43], [44], [45], [46]. Conversely, IL-22, an IL-10-related cytokine, induces gene expression of molecules involved in tissue inflammation, antimicrobial defense and tissue repair and is important for regulating homeostasis of epithelial cells at barrier surfaces [37]. Interestingly, IL-22 promotes innate immunity to bacterial pathogens that directly interact with the host epithelium and induce functional or pathological changes in the epithelium. IL-22 was thus shown to be important for inducing a protective host immunity to the extracellular gram-negative pathogens *K. pneumoniae* in the lung and *C. rodentium* in the intestine [41], [47] but did not have an important role in the host defense against infection with *M. tuberculosis*, *Mycobacterium avium*, *L. monocytogenes* and *S. mansoni* [48], [49].

In conclusion, the development of this set of qPCR primers targeted to key genes involved in the immune response to infection showed that the rabbit ileal loop model of infection by *Shigella* is an accurate model of the disease not only morphologically but also with respect to the gene activation underlying the response; it revealed a clear immunological response at early time points of the infection and it identified the activation of first responder genes and previously unexplored cytokines involved in the innate mucosal immunity to *Shigella*. Thus, this primer set will be highly valuable to characterize the virulence of mutants of *Shigella* and other pathogens in a quantitative manner in this *in vivo* model, especially since many bacterial effectors specifically target the innate immune response of the host [50], [51]. Finally, these twenty-five primer pairs should prove useful to investigators using the rabbit as an animal model for any type of system that elicits an immune response.
